# Proteinuria and influence on monoclonal antibody excretion: a pembrolizumab case report and literature review

**DOI:** 10.1007/s13730-025-01000-6

**Published:** 2025-07-02

**Authors:** T. S. S. Teuntje van Es, B. J. M. Bas Peters, G. Gurbey Ocak, E. A. Elisabeth Kastelijn, S. L. Sabine Croonen, F. C. Floris Loeff, M. P. H. Marcel van den Broek

**Affiliations:** 1 Department of Clinical Pharmacy, St. Antonius Hospital, Koekoekslaan 1, 3435 CM Nieuwegein, The Netherlands; 2 Department of Internal Medicine, St. Antonius Hospital, Nieuwegein, The Netherlands; 3 Department of Pulmonology, St. Antonius Hospital, Nieuwegein, The Netherlands; 4 Department of Pathology, St. Antonius Hospital, Nieuwegein, The Netherlands; 5 Sanquin Diagnostic Services, Amsterdam, The Netherlands; 6 Department of Pharmaceutical Sciences, Utrecht University, Utrecht, The Netherlands

**Keywords:** Nephrotic syndrome, Monoclonal antibodies, Proteinuria, Therapeutic drug monitoring

## Abstract

Therapeutic monoclonal antibodies (mAbs) have revolutionized the treatment landscape of various diseases, offering targeted therapy options with high specificity. Under normal physiological conditions, their size prevents renal excretion. However, there is limited information about mAbs pharmacokinetics in patients with massive proteinuria, a condition often associated with a nephrotic syndrome. In this case report, we describe a 68-year-old man with non-small-cell lung carcinoma (NSCLC) and a paraneoplastic nephrotic syndrome, who was treated with pembrolizumab 200 mg every 3 weeks. Since there is limited data on pembrolizumab disposition in patients with nephrotic syndrome, we monitored pembrolizumab serum and urine concentrations to ensure adequate systemic exposure. Therapeutic drug monitoring results showed no renal excretion of pembrolizumab and therapeutic drug exposure. Treatment of the NSCLC led to an amelioration of the paraneoplastic nephrotic syndrome. We conducted a literature review on the various types of proteinuria and their effects on the excretion of mAbs. Existing literature shows that increased renal clearance of monoclonal antibodies in patients with glomerular proteinuria is possible, but it probably depends on the amount of glomerular proteinuria. Based on literature findings and our own, we suggest that in cases of severe glomerular proteinuria, like nephrotic range proteinuria, the likelihood of renal loss of monoclonal antibodies is higher than in other cases.

## Introduction

Therapeutic monoclonal antibodies (mAbs) have revolutionized the treatment landscape of various diseases, offering targeted therapy options with high specificity. MAbs exhibit unique pharmacological mechanisms of action and pharmacokinetic properties due to their large size and hydrophilic nature. This results in a relatively small volume of distribution and slow distribution throughout the body. Under normal physiological conditions, their size prevents renal excretion, and active neonatal Fc receptor (FcRn)-mediated reabsorption in the proximal tubules takes place [1]. Elimination is primarily through proteolytic catabolism or target-mediated drug disposition, resulting in both linear and non-linear elimination characteristics of mAbs [2].

There is limited information about mAbs pharmacokinetics in patients with massive proteinuria, a condition often associated with a nephrotic syndrome. This syndrome is characterized by significant proteinuria (at least 3.5 g/24 h), hypoalbuminemia (less than 30 g/L) and edema [3]. Nephrotic-range proteinuria is associated with renal clearance of mAbs, as observed in studies with adalimumab and rituximab [4–6]. This raises questions on how proteinuria might influence the pharmacokinetics of other mAbs as well.

In this case report, we describe a patient treated with the PD-1 inhibitor pembrolizumab for non-small-cell lung carcinoma (NSCLC) with nephrotic range proteinuria due to paraneoplastic nephrotic syndrome. We determined the serum and urine concentrations of pembrolizumab during treatment. Furthermore, a comprehensive review of existing literature on the subject is provided, aiming to shed light on the etiology of proteinuria and its implications for mAbs pharmacokinetics.

## Case report

A 68-year-old male has been diagnosed in December 2021 with NSCLC adenocarcinoma of the left lung (cT3N3M1c) and brain metastases. Immunohistochemistry revealed a PD-L1 tumor proportion score of more than 50% (Roche SP263 assay, Ventana Benchmark Ultra). Apart from a KRAS exon 3 mutation, next-generation sequencing and fluorescence in-situ hybridization (FISH) did not identify targets for first-line targeted therapy.

**Fig. 1:**
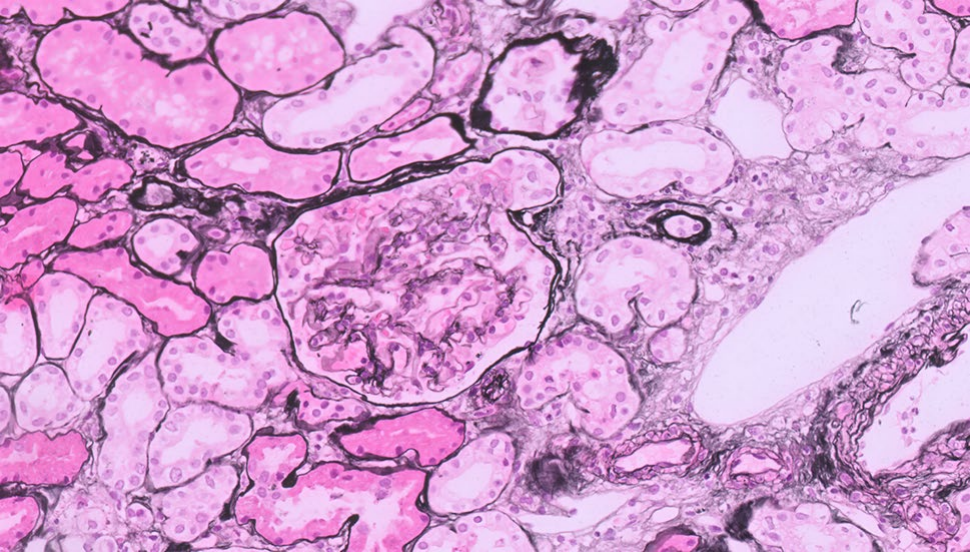
PAMS staining with a glomerulus with slight vacuolation of glomerular basement membrane, without evidence of spikes. IgG showed strong granular positivity along the basement membrane

**Fig. 2:**
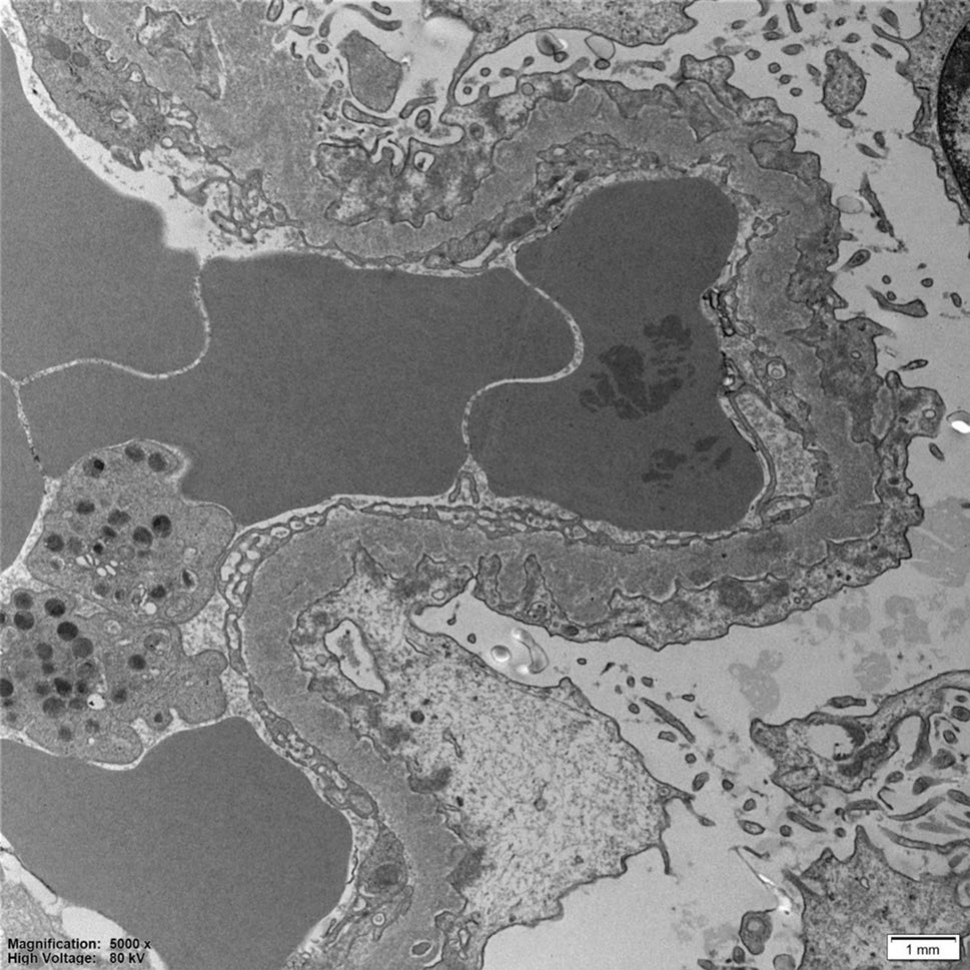
Electron microscopy of a glomerulus with extensive deposits of subepithelial and focally mesangial electron dense amorphous deposits. Diffuse effacement of podocyte foot processes

**Fig. 3:**
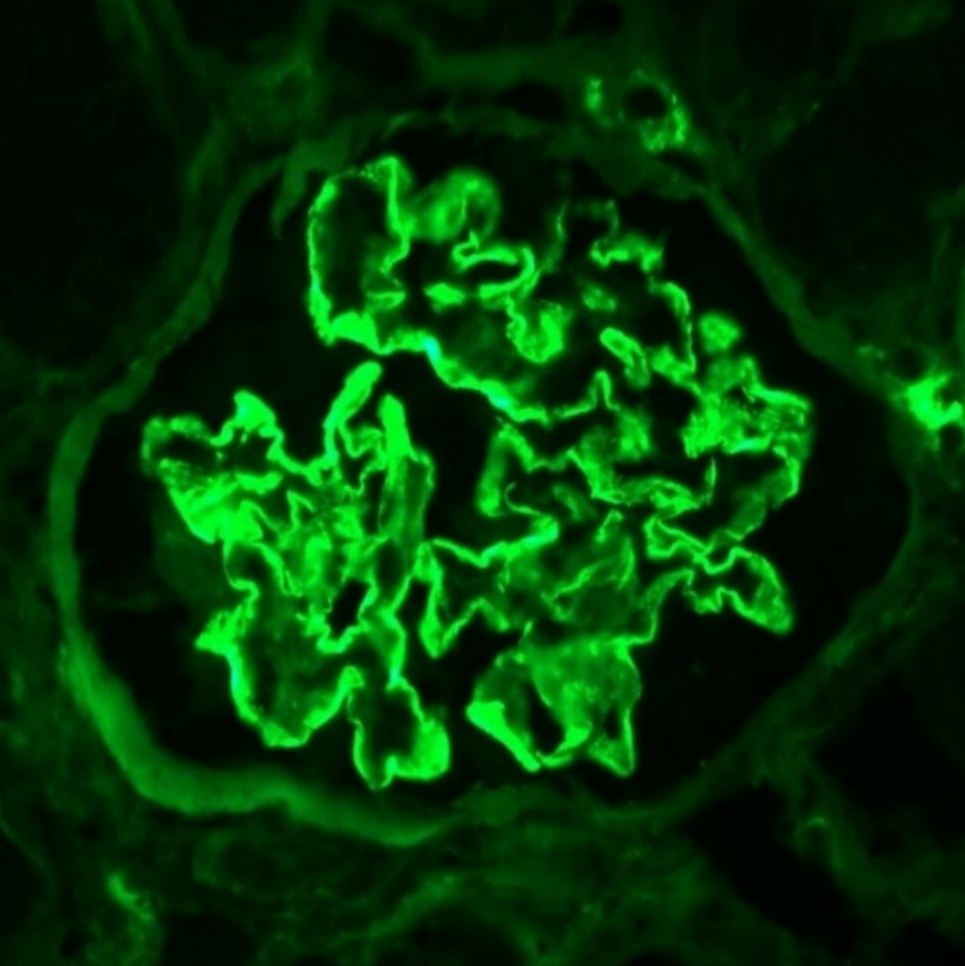
Immunofluorescence IgG staining with granular and partially linear staining along the glomerular basement membrane

The treatment plan consisted of cranial stereotactic radiation followed by pembrolizumab monotherapy. Before initiation of the immunotherapy, the patient was hospitalized because of a *S. pneumoniae* pneumonia (Pneumonia Severity Index V). During admission, edema was noticed for which diuretics were initiated and additional diagnostics were performed. The laboratory results revealed a hypoalbuminemia (12.1 g/L) and proteinuria (urine protein–creatinine ratio (UPCR) 5.3 g/gCr). The estimated glomerular filtration rate (CKD-EPI) was normal (82 ml/min). Other clinical characteristics at the time of diagnosis of nephrotic syndrome are shown in Table 1. Consequently, a kidney biopsy was conducted to investigate the cause of the nephrotic syndrome, leading to the diagnosis of paraneoplastic membranous nephropathy (Figs. 1, 2, 3).

**Table 1:** Clinical characteristics at diagnosis of nephrotic syndrome

Age	68
Gender	Male
Body weight	54 kg
Body mass index	20.3 kg/m^2^
Hematology	
Hemoglobin	7.4 mmol/L
Hematocrit	0.34 L/L
MCV	85 fL
Platelets	517*10^9^/L
Leukocytes	11.5*10^9^/L
Liver test	
Aspartate aminotransferase	45 U/L
Alanine aminotransferase	71 U/L
Alkaline phosphatase	150 U/L
γGT	212 U/L
Total bilirubin	4 µmol/L
Sodium	135 mmol/L
Potassium	4.3 mmol/L
Glucose	6.2 mmol/L
Albumin	12.1 g/L
C-reactive protein	132 mg/L
Serum creatinine	84 umol/L
eGFR (CKD-EPI)	82 ml/min/1.73m^2^
Urea	12.6 mmol/L
Urine albumin–creatinine ratio	144.6 mg/mmol
Urine protein–creatinine ratio (UPCR)	5.3 g/gCr
Urine protein	5.81 g/L
Relevant medication	Hydrochlorothiazide 12.5 mg OD Amlodipine 5 mg OD Olmesartan 20 mg OD

Antiphospholipase A2 receptor autoantibodies were absent. Paraneoplastic membranous nephropathy is a manifestation of malignancy unexplained by direct tumor burden, the extent of invasion or a metastatic process of the disease. Membranous nephropathy may be associated with malignancies that are primarily solid tumors of the lung, prostate, and gastrointestinal tract [7]. Paraneoplastic membranous nephropathy was suspected since there was no apparent alternative etiology available to explain the clinical findings. Pembrolizumab treatment was initiated and it was hypothesized that treatment of the adenocarcinoma would mitigate paraneoplastic nephrotic effects. The patient, with a body weight of 54 kg, received a dose of 200 mg pembrolizumab every 3 weeks.

Since there is limited data on pembrolizumab disposition in patients with nephrotic syndrome, we monitored pembrolizumab serum and urine concentrations to ensure adequate systemic exposure. On day one of cycle one, 24-h urine was collected for protein, creatinine, and pembrolizumab urinalysis. In addition, a blood sample was drawn at the end of the pembrolizumab administration to determine the serum peak concentration. Between day 14–20 of cycle one, urine collection was repeated for pembrolizumab urinalysis and a blood sample was drawn for the determination of a pembrolizumab through concentration. The same sampling schedule was repeated after the third pembrolizumab dose. Pembrolizumab concentrations in serum and urine were quantified using an ELISA method developed by Sanquin Diagnostics Services and validated following the U.S. Food and Drug Administration guidelines [8].

The results for serum and urine drug concentrations are shown in Table 2. Clinical parameters for nephrotic syndrome during treatment with pembrolizumab are shown in Table 3. Pembrolizumab was not detected in the 24-h urine collection sample. The 24-h urine contained 1.12 g of protein.

**Table 2:** Serum and urine concentrations of pembrolizumab

	After first dose	After third dose
Pembrolizumab serum peak concentration (ug/mL)	74.2	79.9
Pembrolizumab serum through concentration (ug/mL)	4.8	13.1
Pembrolizumab urine concentration (ug/mL)	< 0.100	< 0.100

**Table 3:** Clinical parameters for nephrotic syndrome during treatment with pembrolizumab

	Diagnosis nephrotic syndrome	C1D1	C1D14-20	C2D7	C3D14-20
Serum creatinine (µmol/L)	84	52	64	75	62
Serum albumin (g/L), ref. > 35 g/l	12.1	19.8	25.6	–	29.1
Urine protein (g/L), ref. < 0.15 g/L	5.81	1.76	–	1.2	1.17_1_
Urine protein–creatinine ratio (g/gCr)	5.3	3.2	–	3.4	3.3_1_

1 Exception: measured on C3D7

Ambulatory follow-up showed a prompt improvement in symptoms of edema after initiating loop diuretics. Throughout pembrolizumab treatment, serum albumin improved and remained stable around 34–38 g/L, 4–8 months after initiation of pembrolizumab treatment. Proteinuria decreased but remained present throughout treatment with pembrolizumab (UPCR 2.7 g/gCr after 5 months and 0.7 g/gCr after 9 months of pembrolizumab therapy). Tumor response evaluation after cycle four showed reduction of both target and non-target lesions (partial response) according to RECIST 1.1 criteria. After 6 months of therapy, the pembrolizumab dose was changed from 200 mg fixed dose to 4 mg/kg every 6 weeks according to a change in the national dosing guidelines. In September 2022, progression of bone metastases was observed for which radiation was performed. Two months later, progression of the target lesion of the right upper lobe of the lung was seen after which pembrolizumab treatment was discontinued and second-line treatment carboplatin with pemetrexed was initiated. The patient had been treated for a total of 9 months with pembrolizumab until progression occurred.

## Discussion

In our patient with NSCLC adenocarcinoma, brain metastases and a nephrotic syndrome due to a secondary membranous nephropathy treated with pembrolizumab, no renal excretion of pembrolizumab could be detected. After three doses, the pembrolizumab peak plasma concentration reached 79.9 ug/mL, with a trough of 13.1 ug/mL. This aligns with model-based pharmacokinetics predictions by Ahamadi et al., who forecasted a trough concentration of 16 ug/mL after three doses of 2 mg/kg every 3 weeks [9]. Of note, target saturation of PD-1 inhibition by pembrolizumab is already achieved at trough concentrations of 1 ug/mL and above [10, 11]. Thus, based on these findings, we assume target saturation in our patient. In addition, undetectable pembrolizumab concentrations in urine suggest that the paraneoplastic nephrotic syndrome did not negatively affect drug exposure. With treatment, clinical indicators of nephrotic syndrome improved since serum albumin increased from 19.8 to 29.1 g/L and urine protein declined from 1.76 to 1.17 g/L over the initial three cycles. However, the UPCR remained unchanged in this period.

Jansen et al. reported on a similar case receiving pembrolizumab for a NSCLC who also had a paraneoplastic nephrotic syndrome [12]. In their patient, also a therapeutic pembrolizumab trough concentration (73.1 ug/mL, after three doses) was observed. However, in contrast to our findings, pembrolizumab was observed in their patient’s urine in a concentration of 2.4 ug/mL. The authors concluded that despite renal loss of pembrolizumab because of the paraneoplastic nephrotic syndrome, treatment resulted in therapeutic drug exposure and both a radiological and clinical response [12]. In nephrotic syndrome, alteration of the glomerular filtration barrier leads to increased urinary excretion of proteins. The severity of this membrane dysfunction correlates with the clinical severity of nephrotic syndrome, as reflected in laboratory findings such as increased proteinuria and decreased serum albumin concentrations [13, 14]. Compared with the case presented in the article by Janssen et al., our patient had a less severe form of paraneoplastic nephrotic syndrome (hypoalbuminemia 19.8 g/L versus 7.0 g/L) and proteinuria (UPCR 3.2 versus 11.5 g/gCr). This is a possible explanation for the difference in urinary pembrolizumab concentrations. However, to our knowledge, there is only scarce evidence regarding studies investigating the renal excretion of pembrolizumab in patients with proteinuria. Looking into the evidence for Immunoglobulin G (IgG), which is also a high-molecular-weight (HMW) protein, there is abundant evidence indicating that increased urine IgG concentrations in nephrotic patients could reflect activity and severity of the glomerulonephritis [13]. Recent clinical studies showed that in patients with glomerular diseases, the urinary excretion of some HMW proteins (immunoglobulins G and M) and of some low-molecular-weight proteins correlates with the severity of the histologic lesions [14]. Based on these findings, we suggest that the degree of glomerular size-barrier dysfunction reflects urinary excretion of HMW proteins, like monoclonal antibodies.

Although paraneoplastic nephrotic syndrome might result in urinary loss of the monoclonal antibody, therapeutic drug exposure was observed in this study and by others previously [12]. Different etiologies of proteinuria could be associated with differences in monoclonal antibody pharmacokinetics. In general, there are four basic etiologies of proteinuria; glomerular, tubular, overflow and post-renal proteinuria. These forms and the effect on immunoglobulin exposure will be discussed below consecutively.

Glomerular proteinuria mostly results from (chronic) hypertension and/or prolonged poor glycemic control. It is characterized by increased filtration of macromolecules across the glomerular capillary wall caused by damage to the glomerular basement membrane and podocytes [15, 16]. In these cases, the glomerular damage is often irreversible. In a patient with atypical hemolytic uremic syndrome (aHUS), variability of eculizumab pharmacokinetics was seen [17]. This was probably due to an increase in urinary drug loss as a result of progression of proteinuria. UPCR reflects urinary leakage of both intermediate MW proteins (e.g., albumin) and HMW proteins. In parallel to the increase in UPCR and drug clearance, a decline in IgG serum concentration was measured. As the chronic kidney disease progressed, proteinuria progressed [17]. The same authors investigated the effect of proteinuria on the pharmacokinetics of eculizumab in patients with aHUS. They predict that the non–target-mediated clearance in case of severe proteinuria (defined as a UPCR of > 3.1 g/gCr) will increase with 42%. UPCR was a relevant covariate for eculizumab clearance. They conclude that patients with severe proteinuria are more at risk for inadequate therapy compared with patients without proteinuria. However, this study only measured eculizumab concentrations in serum, not in urine [18]. Wehling et al. measured eculizumab concentrations in urine before and after eculizumab dosing. A significant correlation between urine eculizumab concentrations and degree of proteinuria was observed [19]. These findings indicate that severe proteinuria affects the pharmacokinetics of monoclonal antibodies, leading to urinary loss and underexposure.

Glomerular proteinuria as a result of a nephrotic syndrome is less common. Most cases of nephrotic syndrome appear to be caused by primary kidney diseases, whereas secondary causes include auto-immune diseases, malignancies or infection [3, 20]. In these examples, proteinuria may be reversed when the underlying illness is treated. Primary membranous nephropathy (pMN) and focal segmental glomerular sclerosis (FSGS) are examples of glomerular auto-immune diseases resulting in glomerular proteinuria. Studies on the pharmacokinetics of the immunosuppressive monoclonal antibodies adalimumab and rituximab, which are used in these patients, reported a lower systemic exposure in case of glomerular proteinuria. Roberts et al. observed enhanced renal clearance of adalimumab in patients with primary FSGS and nephrotic range proteinuria with up to 13% of the dose being excreted in the urine. A non-linear relationship between proteinuria and renal clearance was observed, with increase of renal clearance at UPCR of 12 g/gCr [4]. In addition, the FONT study group observed that the adalimumab exposure in these patients was about halved due to increased clearance. There was an association between the adalimumab serum half-life and the urinary protein–creatinine ratios and serum albumin concentrations. Serum half-life was shorter in FSGS patients who exhibited higher UPCRs and lower serum albumin levels, reflecting enhanced renal clearance [5]. Rituximab exposure in pMN patients was also observed to be lower, with even one study reporting that 56% of their patients had undetectable rituximab plasma concentrations at 3 months potentially due to renal loss. This negatively impacted achieving a clinical remission at 6 months. Enhanced clearance, shorter half-life, and lower exposures were demonstrated in patients with MN. Linear regression analyses demonstrated significant negative relationships between rituximab half-life and urinary protein excretion. This demonstrated that rituximab has an altered pharmacokinetic profile in patients with MN as compared to patient populations without kidney diseases [6, 21, 22].

In summary, evidence from pharmacokinetic studies of eculizumab, rituximab, and adalimumab in patients with glomerular proteinuria indicate that higher UPCRs correlate with shorter serum half-life, enhanced clearance, and lower exposure of monoclonal antibodies. All patients in the aforementioned studies had severe proteinuria (UPCR > 3.1 g/g or > 3.5 g/day). Since only a few studies have measured mAb concentrations in urine, it is challenging to estimate the variability in renal clearance or detection rates in urine based on different types of monoclonal antibodies. Inter-mAb variability in the efficiency of FcRn-mediated reabsorption has been observed, likely due to differences in the physicochemical properties of the variable regions [23]. In addition, the urine matrix may affect the detection efficiency of monoclonal antibodies, as matrix effects on assays have been reported for urinary albumin, and urea is known to exert a denaturing influence on mouse monoclonal antibodies [24, 25]

Tubular proteinuria is characterized by a diminished proximal tubular reabsorption of low-molecular-weight proteins (< 25 kDa). Under normal circumstances, small proteins can be filtered across the glomerulus and are almost completely reabsorbed in the proximal tubule. Tubulointerstitial diseases can lead to increased excretion of these smaller proteins [26–28]. Monoclonal IgG-based antibodies have a size of about 150 kDa. One study observed a very slight increased IgG proteinuric rate in patients with tubular proteinuria [29], to probably no clinically relevant extent. In tubular proteinuria, there is no need to anticipate loss of protein through the urine. Investigating the tubular reabsorption mechanism of intermediate (albumin) and high-molecular-weight proteins (IgA, IgG, mAbs) reveals that this process is mediated by the FcRn, along with other receptors, in the proximal tubules. In cases of an increased filtered load of these proteins across the glomerular membrane, along with defects or saturation of the FcRn, there may be an increase in their urinary elimination. The extent to which this affects the clearance of mAbs remains unclear [15].

Overflow proteinuria is also characterized by increased excretion of low-molecular-weight proteins but due to a marked overproduction of a particular protein exceeding the normal proximal reabsorption capacity. These proteins are often immunoglobulin light chains (i.e., Bence Jones proteins), in multiple myeloma but also lysozyme, myoglobin or free hemoglobin can be seen [30]. Also in this type of proteinuria, there is no need to anticipate loss of protein through the urine to a clinically relevant extent.

Finally, post-renal proteinuria is caused by inflammation of the urinary tract. Only small amounts of IgA and IgG are excreted (< 1 g/day), clinically not relevant [16, 31]. In post-renal proteinuria, there is no need to anticipate loss of protein through the urine.

To conclude, an effect on the pharmacokinetics of monoclonal antibodies is observed only in glomerular proteinuria, particularly in cases of nephrotic range proteinuria.

Importantly, aside from enhanced renal clearance, various other factors affect the pharmacokinetics of mAbs, with binding to available target (i.e., target-mediated drug disposition) being the most predominant factor [32]. Also, hypoalbuminemia is thought to be a marker for elevated protein turnover secondary to a cancer-associated chronic state of systemic inflammation. A higher endogenous catabolic rate for albumin correlates strongly with the catabolic turnover of IgG molecules such as mAbs [33]. Besides hypoalbuminemia being the result of a nephrotic syndrome, it can also be caused by a higher endogenous catabolic rate.

In conclusion, our patient, diagnosed with NSCLC and presenting a paraneoplastic nephrotic syndrome with mild nephrotic range proteinuria, did not exhibit renal loss of pembrolizumab. This was likely due to the mild nephrotic range proteinuria (UPCR 3.2 g/gCr). Measuring serum and urine drug concentrations provided valuable insights, indicating sufficient drug exposure. In patients with a paraneoplastic nephrotic syndrome, addressing the underlying cancer leads to improvements in nephrotic syndrome symptoms, as corroborated by the findings of Jansen et al. and our own findings. If possible, Therapeutic Drug Monitoring of the monoclonal antibody could be used to assure adequate drug exposure in these patients. Existing literature shows that increased renal clearance of monoclonal antibodies in patients with glomerular proteinuria is possible, but it probably depends on the amount of glomerular proteinuria. We suggest that in cases of severe glomerular proteinuria, like nephrotic range proteinuria, the likelihood of renal loss of monoclonal antibodies is higher than in other cases.

## References

[1] Sand KMK, Bern M, Nilsen J, Noordzij HT, Sandlie I, Andersen JT. Unraveling the interaction between FcRn and albumin: opportunities for design of albumin-based therapeutics. Front Immunol. 2014;5:682. 25674083 10.3389/fimmu.2014.00682 PMC4306297

[2] Keizer RJ, Huitema ADR, Schellens JHM, Beijnen JH. Clinical pharmacokinetics of therapeutic monoclonal antibodies. Clin Pharmacokinet. 2010;49(8):493–507. 20608753 10.2165/11531280-000000000-00000

[3] Höppener P, Kleijkers S, Frenken L. Het nefrotisch syndroom bij volwassenen: niet te missen. Huisarts Wet. 2021;7:1–4.

[4] Roberts BV, Susano I, Gipson DS, Trachtman H, Joy MS. Contribution of renal and non-renal clearance on increased total clearance of adalimumab in glomerular disease. J clin Pharmacol. 2013;53(9):919–24. 23813330 10.1002/jcph.121

[5] Joy MS, Gipson DS, Powell L, MacHardy J, Jennette JC, Vento S, et al. Phase 1 trial of adalimumab in focal segmental glomerulosclerosis (FSGS): II. Report of the FONT (Novel therapies for resistant FSGS) study group. Am J Kidney Dis. 2010;55(1):50–60. 19932542 10.1053/j.ajkd.2009.08.019 PMC2804955

[6] Teisseyre M, Cremoni M, Boyer-Suavet S, Ruetsch C, Graca D, Esnault VLM, et al. Advances in the management of primary membranous nephropathy and rituximab-refractory membranous nephropathy. Front Immunol. 2022;13: 859419. 35603210 10.3389/fimmu.2022.859419 PMC9114510

[7] Khan MB, Kaur A, Ali A, Boris A, Spitalewitz S. Complete resolution of paraneoplastic membranous nephropathy following curative therapy of triple-negative breast cancer. Cureus. 2021;13(9):18125. 10.7759/cureus.18125 PMC8528167 34692335

[8] de Vries F, Smit AAJ, Wolbink G, de Vries A, Loeff FC, Franssen EJF. Case report: pharmacokinetics of pembrolizumab in a patient with stage IV non–small cell lung cancer after a single 200 mg administration. Front Oncol. 2022;12: 960116. 36713570 10.3389/fonc.2022.960116 PMC9875126

[9] Ahamadi M, Freshwater T, Prohn M, Li CH, de Alwid DP, de Greef R, et al. Model-based characterization of the pharmacokinetics of pembrolizumab: a humanized Anti–PD-1 monoclonal antibody in advanced solid tumors. CPT Pharmacometrics Syst Pharmacol. 2017;6(1):49–57. 27863186 10.1002/psp4.12139 PMC5270291

[10] Patnaik A, Kang SP, Rasco D, Papadopoulos KP, Elassaiss-Schaap J, Beeram M, et al. Phase I study of pembrolizumab (Mk-3475; anti-Pd-1 monoclonal antibody) in patients with advanced solid tumors. Clin Cancer Res. 2015;21(19):4286–93. 25977344 10.1158/1078-0432.CCR-14-2607

[11] Elassaiss-Schaap J, Rossenu S, Lindauer A, Kang SP, De Greef R, Sachs JR, et al. Using model-based “learn and confirm” to reveal the pharmacokinetics-pharmacodynamics relationship of pembrolizumab in the keynote-001 trial. CPT Pharmacometrics Syst Pharmacol. 2016;6:21–8. 27863143 10.1002/psp4.12132 PMC5270295

[12] Jansen AME, Sriram JD, Pluim D, Maas RJH, Groningen H, Piet B, et al. Therapeutic exposure and successful response to pembrolizumab in a patient with non-small-cell lung cancer despite significant renal loss due to paraneoplastic nephrotic syndrome. Clin Lung Cancer. 2021;22(2):220–3. 10.1016/j.cllc.2020.09.021 33189593

[13] Bazzi C, Rizza V, Casellato D, Tofik R, Berg A, Gallieni M, et al. Fractional excretion of IgG in idiopathic membranous nephropathy with nephrotic syndrome: a predictive marker of risk and drug responsiveness. BMC Nephrol. 2014;15:74. 24886340 10.1186/1471-2369-15-74 PMC4018618

[14] D’Amico G, Bazzi C. Pathophysiology of proteinuria. Kidney Int. 2003;63(3):809–25. 12631062 10.1046/j.1523-1755.2003.00840.x

[15] Chadha GS, Morris ME. Monoclonal antibody pharmacokinetics in type 2 diabetes mellitus and diabetic nephropathy. Curr Pharmacol Rep. 2016;2:45–56.

[16] Rose BD. Pathophysiology of renal disease. 2nd ed. New York: McGraw-Hill; 1987. p. 11.

[17] Bouwmeester RN, Ter Avest M, Wijnsma KL, Duineveld C, Ter Heine R, Volokhina EB, et al. Case report: variable pharmacokinetic profile of eculizumab in an aHUS patient. Front Immunol. 2021;11: 612706. 33519821 10.3389/fimmu.2020.612706 PMC7843372

[18] Ter Avest M, Steenbreker H, Bouwmeester RN, Duineveld C, Wijnsma KL, van den Heuvel LPWJ, et al. Proteinuria and exposure to eculizumab in atypical hemolytic uremic syndrome. Clin J Am Soc Nephrol. 2023;18(6):759–66. 36913245 10.2215/CJN.0000000000000145 PMC10278783

[19] Wehling C, Amon O, Bommer M, Hoppe B, Kentouche K, Schalk G, et al. Monitoring of complement activation biomarkers and eculizumab in complement-mediated renal disorders. Clin Exp Immunol. 2016;187(2):304–15. 27784126 10.1111/cei.12890 PMC5217898

[20] Kodner C. Nephrotic syndrome in adults: diagnosis and management. Am Fam Physician. 2009;80(10):1129–34. 19904897

[21] Fogueri U, Cheungapasitporn W, Bourne D, Fervenza FC, Joy MS. Rituximab exhibits altered pharmacokinetics in patients with membranous nephropathy. Ann Pharmacother. 2019;53(4):357–63. 30293439 10.1177/1060028018803587 PMC6629258

[22] Teisseyre M, Cremoni M, Boyer-Suavet S, Crepinb T, Benzaken S, Zorzi K, et al. Rituximab immunomonitoring predicts remission in membranous nephropathy. Front Immunol. 2021;12: 738788. 34721403 10.3389/fimmu.2021.738788 PMC8548826

[23] Bryniarski MA, Tuhin MTH, Acker TM, Dl W, Sethaputra PG, Cook KD, et al. Cellular neonatal Fc receptor recycling efficiencies can differentiate target-independent clearance mechanisms of monoclonal antibodies. J Pharm Sci. 2024;113(9):2879–94. 38906252 10.1016/j.xphs.2024.06.013

[24] Sviridov D, Hortin GL. Urine albumin measurement: effects of urine matrix constituents. Clin Chim Acta. 2009;404(2):140–3. 19332047 10.1016/j.cca.2009.03.034

[25] Li Y, Liu M, Kong Y, Guo L, Yu X, Yu W, et al. Significantly improved detection performances of immunoassay for ractopamine in urine based on highly urea-tolerant rabbit monoclonal antibody. Food Chem Toxicol. 2022;168: 113358. 35964837 10.1016/j.fct.2022.113358

[26] Carter JL, Tomson CR, Stevens PE, Lamb EJ. Does urinary tract infection cause proteinuria or microalbuminuria? A systematic review Nephrol Dial Transplant. 2006;21(11):3031–7. 16861738 10.1093/ndt/gfl373

[27] Portman RJ, Kissane JM, Robson AM. Use of beta 2 microglobulin to diagnose tubulo-interstitial renal lesions in children. Kidney Int. 1986;30(1):91–8. 3528618 10.1038/ki.1986.156

[28] Sesso R, Santos AP, Nishida SK, Klag MJ, Carvalhaes JT, Ajzen H, et al. Prediction of steroid responsiveness in the idiopathic nephrotic syndrome using urinary retinol-binding protein and beta-2-microglobulin. Ann Intern Med. 1992;116(11):905–9. 1580447 10.7326/0003-4819-116-11-905

[29] Waldmann TA, Strober W, Mogielnicki RP. The renal handling of low molecular weight proteins: II. Disorders of serum protein catabolism in patients with tubular proteinuria, the nephrotic syndrome, or uremia. J Clin Invest. 1972;51(8):2162–74. 5054468 10.1172/JCI107023 PMC292373

[30] Barratt J, Topham P. Urine proteomics: the present and future of measuring urinary protein components in disease. CMAJ. 2007;177(4):361–8. 17698825 10.1503/cmaj.061590 PMC1942114

[31] Guder WG, Hofmann W. Differentiation of proteinuria and haematuria by single protein analysis in urine. Clin Biochem. 1993;26(4):277–82. 7694813 10.1016/0009-9120(93)90125-p

[32] Oude Munnink TH, Henstra MJ, Segerink LI, Movig KLL, Brummelhuis-Visser P. Therapeutic drug monitoring of monoclonal antibodies in inflammatory and malignant disease: translating TNF-a experience to oncology. Clin Pharmacol Ther. 2016;99(4):419–31. 26265133 10.1002/cpt.211

[33] Ryman JT, Meibohm B. Pharmacokinetics of monoclonal antibodies. CPT Pharmacometrics Syst Pharmacol. 2017;6(9):576–88. 28653357 10.1002/psp4.12224 PMC5613179

